# Child behaviors associated with childhood obesity and parents’ self-efficacy to handle them: Confirmatory factor analysis of the Lifestyle Behavior Checklist

**DOI:** 10.1186/s12966-015-0194-4

**Published:** 2015-03-11

**Authors:** Anna Ek, Kimmo Sorjonen, Jonna Nyman, Claude Marcus, Paulina Nowicka

**Affiliations:** Division of Pediatrics, B62, Department of Clinical Science, Intervention and Technology, Karolinska Institutet, 141 86 Stockholm, Sweden; Division of Psychology, Department of Clinical Neuroscience, Karolinska Institutet, 171 65 Solna, Sweden

**Keywords:** Lifestyle behavior checklist, Children, Eating, Obesity, Parenting, Validation, Confidence

## Abstract

**Background:**

The development of family-based programs for child weight management requires an understanding of parents’ difficulties in managing children’s eating and physical activity behaviors; however, knowledge about the specific behaviors that parents find most difficult to address is still limited. The Lifestyle Behavior Checklist (LBC) is an Australian instrument that assesses parents’ perceptions of children’s obesity-related behaviors (the Problem scale), and parents’ self-efficacy in dealing with these behaviors (the Confidence scale). Our aims were 1) to examine the psychometric properties (the factor structure, internal reliability, construct and discriminative validity) of the LBC in parents of preschoolers in Sweden, using the Child Feeding Questionnaire (CFQ) as a criterion measure, 2) to study associations between the LBC and socio-demographic factors.

**Methods:**

The LBC and the CFQ (measuring parental feeding practices) were distributed to parents from 25 schools/preschools and to parents starting a childhood obesity intervention. To test the fit of the original four-factor model (*misbehavior in relation to food*, *overeating*, *emotional correlates of being overweight, physical activity* (24 items)) to the data, confirmatory factor analysis (CFA) was performed. Structural equation modelling was used to examine associations between the LBC and the CFQ and socio-demographic factors.

**Results:**

In a sample of 478 parents, a five-factor structure proved best fit to data, after excluding 6 items and allowing two pairs of error terms to correlate (TLI = 0.899; CFI = 0.918; RMSEA = 0.042; SRMR = 0.055). The Confidence scale indicated unidimensionality, therefore a hierarchical CFA with 5 first order factors and one second order factor was tested showing good fit. The validity of the LBC was proven by relevant associations with the CFQ and child weight status; parental responses differed depending on child weight status. The Confidence scale was not associated with any child or parent variables.

**Conclusions:**

In a large sample of Swedish parents of preschoolers, the LBC showed good psychometric properties, with relevant correlations to similar constructs. A five-factor structure showed best fit to data with moderate to high internal reliability. The LBC was shown to discriminate effectively between parents of normal weight children and parents of overweight/obese children.

## Background

While parents of young children ably respond to various challenges, childhood obesity is one problem which few parents feel capable of managing [1-3]. The development of effective family-based programs for child weight management requires a better understanding of parents’ difficulties in managing children’s eating and physical activity behaviors; however, knowledge about the specific behaviors that parents find most difficult to address is still limited [4]. Systematic research has been hindered by a lack of user-friendly (short and simple) instruments that capture children’s problematic behaviors related to food and physical activity and parents’ capacity to handle them [5].

The Lifestyle Behavior Checklist (LBC) is a user-friendly instrument designed to assess parents’ perceptions of children’s problematic behaviors related to overweight and obesity regarding eating, physical activity, screen time and overweight (the Problem scale), and parents’ self-efficacy in dealing with these behaviors (the Confidence scale) [1,6]. The instrument was developed in Australia in a systematic way, on the basis of interviews with child obesity experts, observations, and feedback from parents participating in a parenting program [1,6]. In two Australian studies, the LBC was tested for its content validity, factor structure, internal reliability and test-retest reliability [1,6]; the construct validity was tested with criterion measures of general parenting [6]. In a sample of children aged 4-11 years, the LBC was proven to distinguish effectively between parents of children with overweight or obesity and parents of children with normal weight, with the former group scoring higher on the Problem scale and lower on the Confidence scale [1,7]. The LBC has also been used in the evaluation of an obesity treatment program for children 4-11 years old, and has been shown to capture changes in both the child’s behavior and the parents’ confidence in managing their child’s behavior [8].

A recent preliminary evaluation of the LBC outside Australia, conducted in the Netherlands, tested its internal consistency, construct validity and test-retest reliability, with encouraging results [9]. However, confirmatory factor analysis (CFA) has not yet verified the LBC’s original four-factor structure of *misbehavior in relation to food, overeating, emotional correlates of being overweight* and *physical activity*. Thus, it is still unknown whether the proposed four-factor model would stay unchanged for children in different age groups and from various cultures. Careful validation of the instrument when used in a new target population is important as cultural differences in perceptions of appropriate child behaviors and parenting practices may influence the understanding of the items and thus the interpretation of the results [10-12]. For example, the Child Feeding Questionnaire (CFQ), a widely used instrument that assesses parental feeding practices, had to be adjusted repeatedly as a result of CFA when the items were tested in new populations/countries [13]. When a validation study of the CFQ was conducted among parents of preschoolers in Sweden, parental food restriction practices had the lowest frequencies ever reported, as compared to parents in the United States, Japan and Australia [13].

The aims of this study were:

1. to examine the psychometric properties (the factor structure, internal reliability, construct validity (convergent and discriminant validity) and discriminative validity) of the translated LBC in a Swedish population of parents of preschool-aged children, using the CFQ as a criterion measure.
2. to examine associations between the LBC and socio-demographic factors (child and parental age, gender and weight status, parental educational level and parental country of origin).

The preschool age was chosen because recent research emphasizes the critical need of and effectiveness of interventions early in life [14,15]. We hypothesized that because the previous populations in which the LBC had been tested involved older children, certain questions in the LBC would not be age appropriate for our sample of preschoolers, and therefore the CFA would not show an acceptable fit to the original four-factor model (testing the factor structure). We anticipated that children’s problematic behaviors would be associated with lower parental confidence (testing convergent validity). We also anticipated that certain obesity-related behaviors would be associated with parental concern for child weight and feeding practices, such as restriction and monitoring (testing convergent validity). Regarding associations with socio-demographic factors, we expected positive associations between child weight status and all factors on the LBC problem scale (testing convergent validity). We assumed no or weak associations between the CFQ factor *perceived parent weight* and the LBC factors (testing discriminant validity), in line with the findings of previous research [6,9]. Finally, we expected to find differences between the reports of parents of normal weight children and parents of overweight or obese children (testing discriminative validity).

## Methods

### Description of the Lifestyle Behavior Checklist (LBC)

The LBC consists of 25 items divided on two different scales: the Problem scale and the Confidence scale. The Problem scale assesses parents’ perceptions of children’s obesity related problem behaviors, loading on four factors regarding *misbehavior in relation to food* (e.g. the child yells about food), *overeating* (e.g. the child eats too much)*, emotional correlates of being overweight* (e.g. the child complains about being overweight) and *physical activity* (e.g. the child complains about being physically active). On the Problem scale, parents rate to what extent a behavior is a problem for them, from 1 (not at all) to 7 (very much). On the Confidence scale, parents rate how confident they are in dealing with the problematic behaviors, from 1 (Certain I can’t do it) to 10 (Certain I can do it). If the respondent had not experienced a particular problematic behavior mentioned in the instrument, s/he is asked to assess his/her confidence hypothetically. The scores for the 25 questions are added to create a measure of the extent of lifestyle-specific behavioral problems, and to assess parental self-efficacy relating to specific behavioral problems [1]. The clinical cut-off values for the Problem scale are above 50 (range = 25 to 175) and for the Confidence scale under 204 (range = 25 to 250) [8]; the scores were developed on the basis of a comparison with means from a healthy weight population (community sample) [16].

The LBC has shown high internal reliability in three Australian populations (Cronbach’s alpha; 0.87, 0.93, 0.97 (the Problem scale) and 0.95, 0.97, 0.92 (the Confidence scale)) [1,6,7] and in one Dutch population (Cronbach’s alpha; 0.92 (the Problem scale) and 0.98 (the Confidence scale) [9], and good consistency with other instruments measuring child behavior and parenting [1,5-7,9].

### Criterion measure

To test how the LBC correlates to a validated questionnaire we used the CFQ as a criterion measure [17]. The CFQ assesses parents’ perceptions and concerns about child obesity, as well as their child-feeding attitudes and practices [17]. The instrument is well suited for use in research concerning parents of preschool-aged children [17,18]. The CFQ consists of seven factors. The first four factors measure parents’ perceptions of their own and their child’s weight at different ages, and concerns parents may have that can affect how they control their child’s eating. These four factors are: *perceived responsibility* (3 items), *perceived parent weight* (4 items), *perceived child weight* (3 items) and *concern about child weight* (3 items). The other three factors measure parental attitudes and feeding practices relating to *restriction* (8 items), *pressure to eat* (4 items) and *monitoring* (3 items) [17]. The score for each factor is obtained by calculating a mean score for items loading on that factor. In this study, we used the Swedish version of the CFQ; in a recent population-based validation study, involving parents of preschoolers, this version was demonstrated to have a good fit to data (TLI = 0.95, CFI = 0.94, RMSEA = 0.04, SRMR = 0.05) after excluding two items from the *restriction* factor (both related to using food as reward) [13].

Previous research has shown associations between child obesity related behaviors, parental feeding practices and general parenting [7,19-23]. Therefore, relevant associations between the LBC (measuring child behavior and parenting) and the CFQ (measuring parenting) is evidence for construct validity. Strong to moderate associations will prove convergent validity, while no or weak associations between factors measuring different constructs is evidence for discriminant validity [24]. Correlations between children’s weight status and the LBC factors will prove convergent validity, and differences between the reports of parents of normal weight children and parents of overweight or obese children will prove discriminative validity (i.e. it is possible to discriminate responses between different groups) [24].

### Translation process

The translation process of the LBC was conducted according to standard recommendations [24-26] and in collaboration with the developers of the instrument [1]. The LBC was first translated by two independent translators whose native language was Swedish. The translations were checked for differences and compared with the original version. After discussions between the translators and the research group, a new version of the translated LBC was created. This version was back translated by two other independent translators with no prior knowledge of the original version and whose native language was English. The few differences were due to different choice of wordings such as *grumbles and complains about food* instead of *whinges and whines about food*; in some cases a softer language was chosen as more culturally appropriate for the Swedish context (e.g. *takes food from* others instead of *steals food from others*). To test the comprehensibility of the translated questionnaire, cognitive interviews [27] were performed with five parents (three mothers and two fathers), representing the target population of parents with preschool aged children. Parents were recruited from one preschool and one school in the Stockholm area. In the interviews, the techniques think-aloud and verbal probing were used. When using think-aloud the interviewer asks the respondent to describe how he/she reasons when answering the questions, and with verbal probing the interviewer uses questions to follow-up on the respondent’s answer; both techniques lead to better understanding of the cognitive processes evoked by the questions asked and the answers given [27]. The interviews followed a predefined set of questions, were digitally recorded and lasted for approximately one hour. Further minor adjustments in the choice of wordings and concepts were added after the interviews and incorporated in the final revision of the LBC. We used *the child wants* or *the child asks for* instead of *the child demands* because the parents perceived *demands* as too strong and unsuitable for such a young age group. We changed *steals food from others (e.g. from other children’s lunch boxes)* to *takes food from others (e.g. from family members or other children)* to adapt to the Swedish context, where children do not bring lunch boxes to child-care or school. We also changed *unhealthy snacks* to *unhealthy snack meals* to clarify the meaning of the concept (i.e. includes all meals eaten between the main meals, not just snacks such as potato chips, popcorn, and sweets). Finally, we slightly modified the layout of the questionnaire to clarify to the respondents that there were two scales of the questionnaire to fill out. All these changes were confirmed with the developers of the original instrument.

### Population and data collection

#### School sample

To obtain a representative sample of children in a range of weight categories, the researchers selected schools/preschools from areas with low, medium and high prevalence of obesity, according to data from the most recent primary care report in Stockholm County [28]. School principals and heads of preschools, representing 45 units (30 preschools and 15 schools), were contacted; 20 preschools and 5 schools agreed to participate. A total of 931 parents, 595 parents with children attending preschool and 336 parents with children in the preparation year of school, received the LBC and the CFQ. Completed questionnaires (n = 432; 267 parents of preschoolers and 165 parents of school children) were sent back to the research group in an enclosed envelope. All data were collected anonymously.

#### Clinical sample

To be able to better examine differences between parents of children with overweight and obesity and parents of normal weight children, we added baseline questionnaires from a clinical population of parents (n = 47) participating in a randomized controlled childhood obesity trial for preschoolers (NCT01792531). The children were referred by primary child care centers in Stockholm County.

Both the present study and the clinical study were approved by the Regional Ethical Board in Stockholm (dnr: 2011/1329-31/4, 2012/1104-32, 2012/2005-32, 2013/486-32, 2013/1628-31/2).

### Statistical analysis

The descriptive statistics are presented as means and standard deviations (SD), or numbers and percentages for categorical variables. Independent two-tailed t-tests (for continuous variables) and chi square tests (for categorical variables) were used to report the differences between the school sample and the clinical sample. Independent t-tests were also used to compare group means according to children’s weight status for the LBC’s individual items and scales to assess the discriminative validity of the LBC. All p-values <0.05 were regarded as statistically significant. These analyses, as well as exploratory factor analysis (EFA) and reliability calculations (Cronbach’s alpha), were conducted with SPSS version 22. MPlus version 7.11, using Maximum Likelihood with Robust standard errors (MLR) estimation, was used to perform CFA and structural equation modelling (SEM). EFA was used to replicate the original factor structure and to guide the further testing of the factor structure with CFA.

CFA is recommended to test the factor structure when previous hypotheses about the dimensions of the construct are available based on theory and/or previous analysis [29]. The original four-factor model [6] was tested with CFA to examine fit to the data. If one or more items would not load on the original factors after translation, this would indicate that these items had a different meaning, either due to the translation, or due to poor fit to the target population. To evaluate the fit of the factor structure to our data, we used four commonly recommended fit indices: the comparative fit index (CFI), the Tucker-Lewis Index (TLI), the root mean square error of approximation (RMSEA) and standardized root mean square residual (SRMR). Adequate fit was indicated by CFI and TLI values over 0.90 [30] and good fit was indicated by values over 0.95, a RMSEA of 0.06 or lower and a SRMR of 0.08 or lower [31].

To compare groups according to children’s weight status, weight categories were created using age and gender specific international cut offs for body mass index (BMI) [32,33]. Children with underweight (child weight status equivalent to BMI < 17) were excluded (n = 18) from the analysis as well as from the description of body mass index standard deviation scores (BMI SDS), because these data were not relevant to the purpose of this study. BMI SDS was derived from Swedish age- and sex specific reference values [34].

Structural equation modelling (SEM) analyses were conducted to test the construct validity of the LBC by examining the correlations between the LBC and the CFQ’s factors; SEM analyses were also conducted to examine the associations between the LBC and socio-demographic factors (child characteristics; gender, age, BMI SDS, and parental characteristics; gender, age, BMI, Nordic background and education level).

## Results

### Sample characteristics

The sample characteristics are presented in Table 1. In the total sample (n = 478), 70% of the parents had a university degree, mean parental BMI was 24.0 (SD 3.8); 69% were of normal weight and 31% were overweight (BMI ≥ 25) or obese (BMI ≥ 30). Among the children, 80% were of normal weight, 10% were overweight and 10% were obese.

**Table 1:** **Sample characteristics**

**Variable**	**Total population (n = 478)**		**Clinical sample (n = 47)**		**School sample (n = 431)**		
	**Mean**	**SD**	**Mean**	**SD**	**Mean**	**SD**	**p**
*Continuous*							
Child’s age (years)	5.5	1.0	5.1	0.7	5.5	1.0	<0.001
Parent’s age (years)	38.9	5.0	37.6	7.2	39.0	4.7	0.19
Child BMI SDS	0.2	1.4	3.1	0.7	-0.2	1.0	<0.001
Mother BMI	23.1	3.9	27.6	5.8	23.3	3.3	<0.001
Father BMI	25.5	2.9	26.9	3.7	25.3	2.8	0.11
*Categorical*	**n**	**%**	**n**	**%**	**n**	**%**	
***Child gender***							0.90
Female	249	52	25	53	224	52	
Male	227	48	22	49	205	48	
***Parent gender***							0.65
Female	388	81	37	79	351	81	
Male	90	19	10	21	80	19	
***Country of origin***							<0.001
Nordic	411	87	26	55	385	90	
Non-Nordic	64	13	21	45	43	10	
***Language at home***							<0.001
Swedish	433	91	26	55	407	95	
Other	31	9	21	45	21	5	
***Mother’s education***							<0.001
University degree	274	71	17	46	257	74	
No university degree	111	29	20	54	91	26	
***Father’s education***							0.72
University degree	58	65	6	60	52	66	
No university degree	31	35	4	40	27	34	

*Note* BMI SDS = Body mass standard deviation score. BMI = Body mass index. Country of origin: Nordic = Swedish, Norwegian, Finnish and Danish origin. Mother’s and father’s education: No university degree = high school grad 12 or lower. Independent two tailed t-tests and Chi square tests were used to compare the school sample and the clinical sample with a significance level of p < 0.05.

In the school sample (n = 431), 72% of the parents had a university degree, mean BMI was 23.7 (SD 3.3); 28% were overweight or obese. Thus, as compared to the general population in Stockholm, the parents in the school sample had somewhat higher education and the percentage of overweight/obesity was somewhat lower. Among the children, 9.7% were overweight and 0.3% were obese. In comparison, the prevalence rate of overweight in four-year-olds in Stockholm County in 2013 was 9.4%, while 1.8% of children were classified as obese [28].

In the clinical sample (n = 47), as compared to the school sample, a smaller proportion (49%) of the parents had a university degree and more (60%) of them were classified as overweight or obese. Also, the families were much more ethnically diverse.

### Factor structure and internal reliability

#### The LBC Problem scale

**Figure 1:**
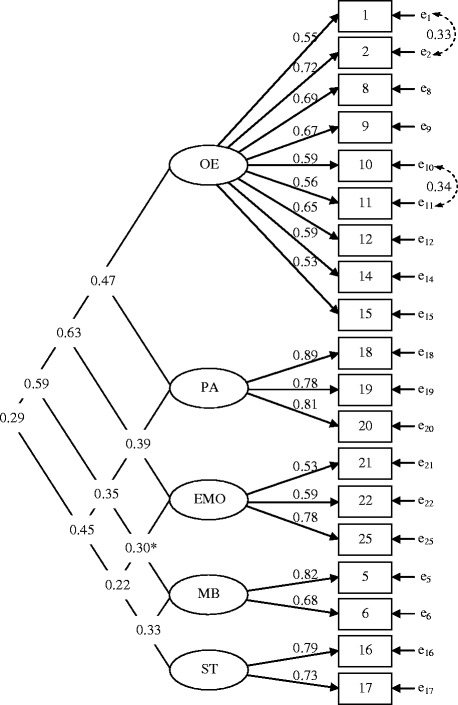
**Confirmatory factor analysis of the Problem scale of the Lifestyle Behavior Checklist.** *Note* The Problem scale of the Lifestyle Behavior Checklist (LBC) with five factors and two added correlations between error terms. The model shows acceptable fit to data, *χ* ^2^(140) = 255, *p* < 0.001; TLI = 0.899; CFI = 0.918; RMSEA = 0.042 (90% CI: 0.033-0.050); SRMR = 0.055. *p < 0.05, otherwise *p* < 0.001. The LBC five order factors in the model are; O*vereating* (OE)*, Physical Activity* (PA), *Emotional correlates of being overweight* (EMO), *Misbehavior in relation to food* (MB) and *Screen Time* (ST). The estimates on the left side in the figure stand for correlations between the factors and the estimates on the right side of the figure stand for factor loadings.

The initial CFA with the original four-factor model by West et al showed a poor fit to the data (TLI = 0.581; CFI = 0.627; RMSEA = 0.079; SRMR = 0.087). EFA of the Problem scale indicated a better fit with a five factor solution (explained variance 52%). After examining factor loadings for specific items, we excluded the items 3, 4, 7, 13, 23 and 24, which improved the model and increased explained variance to 61%. The internal reliability (Cronbach’s alpha) for the total Problem scale (0.85) and for the individual factors was adequate*: overeating* (9 items) 0.82, *physical activity* (3 items) 0.86, *emotional correlates of being overweight* (3 items) 0.65, *misbehavior in relation to food* (2 items) 0.71 and *screen time* (new factor with 2 items) 0.73. CFA of the LBC Problem scale with the modified five factors was conducted. Two pairs of error terms were allowed to correlate and the model showed acceptable fit to data (TLI = 0.899; CFI = 0.918; RMSEA = 0.042; SRMR = 0.055) (Figure 1).

#### The LBC Confidence scale

**Figure 2:**
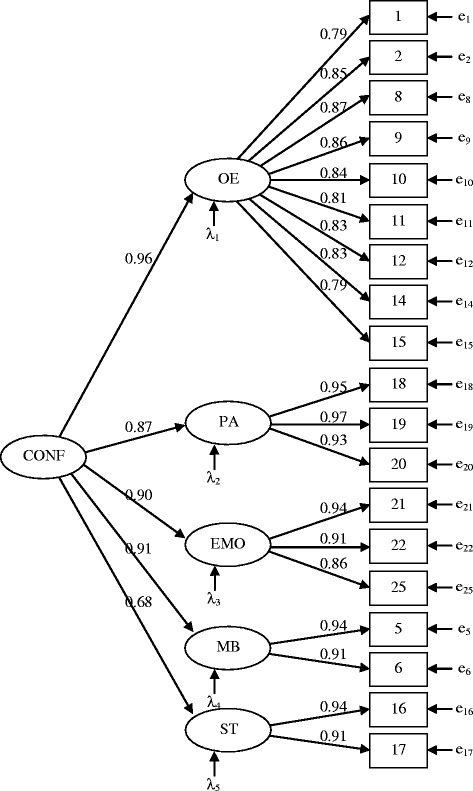
**Confirmatory factor analysis of the Confidence scale of the Lifestyle Behavior Checklist.** *Note* The Confidence scale of the Lifestyle Behavior Checklist (LBC) with five first order and one second order factor. The model shows acceptable fit to data, *χ* ^2^(147) = 427, *p* < 0.001; TLI = 0.927; CFI = 0.937; RMSEA = 0.065 (90% CI: 0.057-0.072); SRMR = 0.042. All parameter values are significant (*p* < 0.001). The LBC five first order factors are: O*vereating* (OE)*, Physical Activity* (PA), *Emotional correlates of being overweight* (EMO), *Misbehavior in relation to food* (MB) and *Screen Time* (ST) and the second order factor is Confidence (CONF). The estimates on the left side of the figure stand for standardized regression coefficients when the first order factors are regressed on the second order factor. The estimates on the right side of the figure stand for factor loadings.

EFA of the Confidence scale indicated unidimensionality which was supported by very high internal reliability (Cronbach alpha 0.98). Furthermore, when the same model as used for the Problem scale was fitted to the Confidence scale, all factors were highly correlated (all *r*s > 0.57). Therefore, a hierarchical CFA with 5 first order factors and one second order factor was tested, showing acceptable fit to data (TLI = 0.927; CFI = 0.937; RMSEA = 0.065; SRMR = 0.042) (Figure 2).

### Validity

A full SEM model, including both the LBC scales and the CFQ factors, was analyzed, with the following modifications: (1) the *perceived child weight* factor of the CFQ was removed, as it created convergence problems; (2) the error terms of the items on the LBC Problem scale were allowed to correlate with the corresponding error terms of the items on the Confidence scale; (3) the factors *overeating* and *misbehavior in relation to food* on the Confidence scale were allowed to correlate, *r* = 0.75, *p* < 0.001; (4) as presented in Figure 1, the error terms for items 1 and 2 as well as 10 and 11 on the Problem Scale were allowed to correlate, and the error terms of items *perceived parent weight 1* and *perceived parent weight 2* on the CFQ were allowed to correlate; (5) non-significant correlations between factors were set to zero. The resulting model showed acceptable fit to data, *χ*^2^(1526) = 2786, *p* < 0.001; TLI = 0.898; CFI = 0.905; RMSEA = 0.042 (90% CI: 0.039-0.044); SRMR = 0.060.

#### Construct validity (convergent and discriminant validity)

The correlations between the LBC and the CFQ factors are presented in Table 2.

**Table 2:** **Correlations between the Lifestyle Behavior Checklist and the Child Feeding Questionnaire**

	**OE**	**PA**	**EMO**	**MB**	**ST**	**CONF**	**PR**	**PPW**	**CN**	**RST**	**PE**	**MN**
Overeating (OE)	-	0.48*	0.59*	0.62*	0.32*	-0.19*	0.09^†^	0.15^†^	0.81*	0.57*	-0.25*	*ns*
Physical activity (PA)		-	0.40*	0.38*	0.52*	-0.43*	*ns*	*ns*	0.31*	0.36*	*ns*	*ns*
Emotional correlates of being overweight (EMO)			-	0.30^†^	0.23*	-0.14^†^	0.22*	*ns*	0.51*	0.43*	*ns*	*ns*
Misbehavior (MB)				-	0.36*	-0.15^†^	*ns*	*ns*	0.37*	0.31*	*ns*	*ns*
Screen time (ST)					-	-0.35*	*ns*	*ns*	0.22*	0.22^†^	*ns*	-0.19*
Confidence scale (CONF)						-	*ns*	*ns*	-0.15^†^	-0.32*	-0.11^†^	*ns*
Perceived responsibility (PR)							-	*ns*	0.20*	0.14^†^	0.15^†^	*ns*
Perceived parent weight (PPW)								-	0.30*	0.19^†^	*ns*	*ns*
Concern about child weight (CN)									-	0.68*	-0.18*	*ns*
Restriction (RST)										-	*ns*	0.19*
Pressure to eat (PE)											-	*ns*
Monitoring (MN)												-

*Note* Correlations between the Lifestyle Behavior Checklist (LBC) factors (O*vereating* (OE)*, Physical Activity* (PA), *Emotional correlates of being overweight* (EMO), *Misbehavior in relation to food* (MB) and *Screen Time* (ST)) and the Confidence scale (CONF) and the Child Feeding Questionnaire (CFQ) factors (*Parental Responsibility* (PR), *Perceived Parent Weight* (PPW), *Concern about child weight* (CN), *Restriction* (RST), *Pressure to Eat* (PE) and *Monitoring* (MN)). The factor *Perceived Child Weight* (PCW) was not included in the model.

*ns* = non-significant = set to zero in the model; ^†^ *p* < 0.05; *p < 0.001.

In summary, parents who scored high on the LBC Problem scale also scored high on the CFQ factors *restriction* and *concern about child weight*. Parents with lower scores for screen time-related problem behaviors reported higher scores on the CFQ factor for *monitoring* of their child’s eating. High scores on the *overeating* factor and the *emotional correlates of being overweight* factor were significantly associated with the CFQ factor *perceived responsibility*. The CFQ factor *perceived parent weight* was significantly correlated only to the LBC factor *overeating* on the Problem scale. The CFQ factor *pressure to eat* was negatively correlated to *overeating* on the LBC Problem scale and to the Confidence scale. High scores on the Problem scale were all correlated with lower confidence in handling obesity-related behaviors. High scores on the Confidence scale were negatively associated with the CFQ factors *concern about child weight*, *restriction* and *pressure to eat*.

#### Discriminative validity

To examine discriminative validity, group means for all the individual items of the Problem scale and the Confidence scale were provided and compared between parents of children with normal weight and parents of children with overweight or obesity (see Table 3). On the Problem scale, 20 of the 25 items significantly differed between the groups. The total scores on the Problem scale for parents of children with normal weight (M = 40.5 (10.1 SD)) were significantly lower (p < 0.001) than those for parents of children with overweight or obesity (M = 53.2 (18.1 SD)). On the Confidence scale, parents of children with overweight or obesity scored significantly lower on 7 of the items. However, no significant difference was observed between the two group’s total scores on the Confidence scale. It has to be noted that the mean values presented in Table 3 were not adjusted for child and parental characteristics, leaving the p-values to indicate how mean values vary depending on child weight status.

**Table 3:** **Group means and differences for the Lifestyle Behavior Checklist’s items and scales**

		**Problem scale**						**Confidence scale**					
		**Normal weight**		**Overweight/ obese**				**Normal weight**		**Overweight/ obese**			
		**(N = 332)**		**(N = 84)**				**(N = 332)**		**(N = 84)**			
**Item** ***my child…***		**M**	**SD**	**M**	**SD**	**F**	**p**	**M**	**SD**	**M**	**SD**	**F**	**p**
1.	Eats too quickly	1.4	0.9	2.6	1.8	117.1	<0.001	8.7	2.1	7.9	2.3	5.1	0.01
2.	Eats too much	1.2	0.7	3.5	2.1	363.9	<0.001	8.6	2.1	7.6	2.3	3.6	<0.001
3.	Eats unhealthy snack meals	1.8	1.1	2.3	1.3	7.9	0.001	8.5	2.1	8.0	2.4	3.3	0.06
4.	Grumbles or complains about food	3.2	1.7	3.1	1.9	1.7	0.47	8.1	2.1	8.1	2.1	0.1	0.99
5.	Shouts about food	1.4	0.9	2.1	1.7	56.5	<0.001	8.7	2.1	8.4	2.2	0.4	0.22
6.	Has anger tantrums about food	1.4	0.9	1.9	1.6	46.9	<0.001	8.5	2.2	8.4	2.2	0.1	0.74
7.	Refuses to eat some foods (i.e. is fussy with food)	3.3	1.8	2.8	1.9	0.3	0.02	7.9	2.3	7.9	2.5	1.0	1.00
8.	Argues about food (e.g. when you say *That’s enough, no more*)	1.4	0.9	2.7	1.8	113.5	<0.001	8.6	2.0	8.1	2.1	0.2	0.02
9.	Wants extra portions at meals	1.7	1.1	3.1	2.0	66.5	<0.001	8.8	2.0	7.9	2.3	6.8	<0.001
10.	Constantly asks for food between meals	1.7	1.0	2.7	1.8	65.7	<0.001	8.7	2.0	7.9	2.3	2.8	0.001
11.	Wants food when you are out shopping or doing other things outside the home	1.8	1.1	2.7	1.8	45.0	<0.001	8.7	1.9	7.9	2.7	12.0	0.002
12.	Sneaks food when they know they are not supposed to	1.1	0.4	1.9	1.5	154.6	<0.001	8.7	2.2	8.1	2.5	4.9	0.03
13.	Hides food	1.0	0.2	1.2	1.0	50.1	<0.001	8.7	2.2	8.3	2.7	6.6	0.17
14.	Takes food from others (e.g. family members or children)	1.0	0.3	1.5	1.2	120.3	<0.001	8.8	2.1	8.5	2.5	2.6	0.19
15.	Eats for comfort when feeling let down or depressed	1.1	0.4	1.6	1.3	119.4	<0.001	8.4	2.4	8.2	2.4	0.001	0.38
16.	Watches too much TV	2.7	1.5	3.2	1.6	0.1	0.006	8.1	2.1	7.8	2.1	0.1	0.20
17.	Spends too much time playing video or computer games.	2.5	1.5	2.5	1.7	0.9	0.76	8.0	2.2	8.3	2.2	0.002	0.42
18.	Complains about having to be physically active (e.g. *This is boring, I’m tired, My leg hurts*)	1.8	1.1	2.6	1.8	60.8	<0.001	8.4	2.1	8.1	2.2	0.3	0.42
19.	Does not want to be physically active	1.5	0.9	1.8	1.4	24.4	0.003	8.4	2.1	8.2	2.3	1.8	0.52
20.	Complains about not having enough energy	1.8	1.1	2.2	1.6	21.3	0.004	8.3	2.1	8.1	2.2	<0.001	0.53
21.	Complains about being overweight	1.0	0.1	1.4	1.0	190.6	<0.001	8.3	2.4	7.7	2.7	1.5	0.6
22.	Complains about being teased	1.2	0.6	1.6	1.2	37.0	<0.001	7.9	2.6	7.5	2.7	0.6	0.24
23.	Complains about not having enough friends	1.3	0.9	1.3	0.8	0.5	0.58	7.8	2.6	7.9	2.5	1.4	0.76
24.	Complains about not looking good	1.1	0.5	1.2	0.6	10.5	0.08	8.1	2.6	8.0	2.7	0.003	0.80
25.	Complains about clothes being too small	1.1	0.4	1.7	1.5	135.5	<0.001	8.6	2.2	8.4	2.3	0.5	0.39
	Total Problem scale score	40.5	10.1	53.2	18.1	63.5	<0.001						
	Total Confidence scale score	210.4	45.8	202.9	41.3	0.5	0.19						

*Note* Independent two tailed t-tests were used to compare group means with a significance level of p < 0.05 for the Lifestyle Behavior Checklist’s (LBC) individual items and for the total score of the Problem and Confidence scales for parents to normal weight and overweight/obese children.

### Associations between the LBC and socio-demographic variables

When child and parental characteristics were analyzed in non-adjusted and adjusted models, the child’s BMI SDS was shown to be most influential as it was significantly and positively associated with all LBC factors on the Problem scale except *screen time* (see Table 4). Among parental characteristics, only BMI was positively associated with the LBC *screen time*. The Confidence scale, on the other hand, was not significantly correlated with any of the studied child or parental variables. The studied background variables explained 37% of the variance for the LBC *overeating* factor and 25% of the variance for *emotional correlates to being overweight*.

**Table 4:** **Standardized effects of child and parental predictors on the Lifestyle Behavior Checklist factors**

	**Overeating**	**Physical activity**	**Emotional correlates of being overweight**	**Misbehavior in relation to food**	**Screen time**	**Confidence**
*Non-adjusted*						
***Child characteristics***						
Age	-0.08	-0.02	0.05	-0.13^†^	0.15^†^	0.07
Girl^a^	0.13	-0.13	0.18^†^	-0.02	-0.36^†^	-0.07
BMISDS	0.59*	0.18^†^	0.45*	0.29*	0.10	-0.12^†^
***Parent characteristics***						
Age	-0.12^†^	-0.04	-0.12^†^	-0.08	-0.02	0.01
Woman^a^	-0.16	-0.34^†^	-0.10	-0.30^†^	0.05	0.09
BMI	0.28*	0.14^†^	0.18^†^	0.09	0.15^†^	-0.08
Nordic^a^	-0.48^†^	-0.23	-0.57^†^	-0.05	-0.37^†^	0.35^†^
Education	-0.10	0.02	-0.17^†^	-0.09	0.01	-0.05
*Adjusted*						
***Child characteristics***						
Age	-0.04	0.03	0.09	-0.10	0.20*	0.03
Girl^a^	0.02	-0.19	0.07	-0.10	-0.37^†^	-0.08
BMISDS	0.54*	0.16^†^	0.40*	0.30*	0.03	-0.07
***Parent characteristics***						
Age	-0.09	-0.06	-0.07	-0.02	-0.01	-0.01
Woman^a^	-0.14	-0.35^†^	0.01	-0.28	0.09	0.09
BMI	0.11	0.07	0.03	-0.00	0.14^†^	-0.07
Nordic^a^	-0.01	-0.15	-0.29	0.31	-0.31	0.26
Education	-0.01	0.05	-0.10	-0.05	0.00	-0.03
*R* ^2^	0.37*	0.07^†^	0.25*	0.12^†^	0.10^†^	0.03

*Note* Standardized effects, both non-adjusted and adjusted for each other, of certain predictors (child characteristics and parental characteristics) on the Lifestyle Behavior Checklist factors: O*vereating* *, Physical Activity*, *Emotional correlates related to being overweight*, *Misbehavior in relation to food*, *Screen Time* and the Confidence scale, as well as *R* ^*2*^ = proportion explained variance (when including all predictors simultaneously)0. *ns* = non-significant = set to zero in the model; ^†^ *p* < 0.05; *p < 0.001; ^a^the predictor is binary, the effects stand for the difference in the outcome, in *SD*, between the two groups0.

## Discussion

This study demonstrates the validity of a modified version of the LBC among parents of preschool-aged children. It shows that the LBC reliably measures parents’ perceptions of child problematic behaviors related to overweight and obesity, as well as parents’ lifestyle-specific self-efficacy in handling these behaviors. The factor analysis suggested that the best fit to the data was obtained with a five-factor model after omitting 6 items. The construct validity of the LBC was proven meaningful with relevant correlations to the CFQ and with a moderate to high internal consistency for both LBC scales. High scores on the Problem scale correlated to lower scores on the Confidence scale. There was also a significant difference in how parents of children with normal weight and parents of children with overweight and obesity responded to the LBC Problem scale, providing further evidence for the discriminative validity of the LBC. Among the examined parent and child characteristics, when adjusted for potential confounders, child BMI SDS was positively associated with the majority of LBC factors, which provides additional evidence that the instrument effectively discriminates obesity-inducing lifestyle behaviors.

### The factor structure of the LBC

In this population of young children a five-factor structure proved to be a better model than the original four-factor model. The new factor, measuring to what extent parents perceive their child’s screen time behaviors as problematic, was part of the physical activity factor in the original model. The introduction of the *screen time* factor in our population is interesting in light of a large meta-analysis on sedentary behaviors showing only a weak association between physical activity and screen time [35], which may imply that physical inactivity and screen time are two different dimensions. The younger age of the children in our sample compared to the previous studies [1,6,9] could explain the relevance of the screen time factor introduced in this study. Younger children are naturally active, more so than older children [36], especially if encouraged by parents [37]. Because parents perceive younger children as active [38], they may perceive increased screen time during the preschool age [39,40] as a more problematic behavior, compared to lack of physical activity.

Six items were omitted to achieve an adequate fit to data in the final model, confirming our hypothesis of the age appropriateness of some questions. Among these were item 13 *(hides food)*, 23 (*complains about not having enough friends)* and 24 (*complains about not being attractive)*, all considered as irrelevant for young children in the cognitive interviews*.* Item 4 (*whinges or whines about food)* was ambiguous, and could mean both that the child wants food and that the child is not happy about the food s/he receives; the model was improved by its exclusion. Also*,* item 7 *(refuses to eat certain food (i.e. fussy eating))* was omitted. In the original four-factor model [6] this question did not load on the expected factor (*misbehavior in relation to food*). Finally, somewhat unexpectedly, item 3 (*eats unhealthy snacks*) also had to be excluded due to the poor fit; however, the problems with this item were already indicated during the process of translation and in the cognitive interviews. Parents understood this item as referring to small planned structured meals between the main meals, often served in kindergarten/preschool, and not to food consumed spontaneously by the child at home; school meals were considered healthy by parents and thus not perceived as problematic.

The factor structure of the Confidence scale has not been examined in earlier studies. In our analyses, we found it somewhat surprisingly to be unidimensional. The unidimensionality means that the scale measures a global self-efficacy of the parent and that this is not specific to certain behaviors or situations. The results suggest that interventions focusing on strengthening any element of parents’ self-efficacy may affect both eating-related and physical activity-related situations. The Australian intervention study, which used the LBC to evaluate a childhood obesity intervention, showed improvements in parental self-efficacy as well as a decrease in child BMI SDS [8].

### Validity

Many significant correlations with the CFQ were seen, supporting our hypotheses and confirming the construct validity of the LBC. As predicted, parents who scored high on the LBC factors also reported being restrictive of their child’s eating. Parental restriction has been positively associated with child weight status [13,20,41]. However, it has not been established if restriction does in fact increase the child’s weight [20,42,43] or if it is a logical response to the child’s overweight. Longitudinal prospective studies on children’s eating behavior and parenting practices around eating are needed to further clarify this process.

Another relevant correlation was *parents’ concern about child weight* with all the LBC factors. As parents are increasingly aware that obesity in childhood is a risk factor for obesity in adulthood, and thus poses risk for serious health consequences later in life [44], it was not surprising to find associations between parents’ concern about their child’s weight and the child’s problematic obesity-related behaviors, indicating that these behaviors may be particularly challenging. Confirming the discriminative validity of the LBC, and in line with the previous studies [1,6,7,9], we also identified a difference in how parents responded to the LBC depending on the child’s weight status. Associations between high scores on the LBC problem factors and low scores on the Confidence scale also imply that experiences of problematic child behavior and failure in handling them affect the self-efficacy of parents, as compared to a parent who never has experienced the behavior but thinks he/she can handle it [6]. Parents’ confidence in handling child problematic lifestyle related behavior is likely to impact the child’s risk of future weight problems [6]. The results signal the importance of providing parents with practical tools in childhood obesity interventions, to help improve the cooperation with their child around healthy lifestyle behaviors [8]. The LBC is an appropriate instrument to use in the evaluation of such interventions.

Interestingly parental monitoring of children’s eating was correlated with low scores for screen time problematic behavior. A recent US study has reported how maternal monitoring of preschoolers’ media time was associated with lower child BMI SDS [45]. Together the results suggest that monitoring is an important parenting practice for promoting a healthy lifestyle for children [22,23].

To test the discriminant validity of the LBC, we expected no or low correlations to the CFQ factor measuring how parents perceive their own weight. Only the LBC *overeating* factor was associated with the CFQ factor. A possible explanation for this association is that overweight or obese parents with his/her own overeating experiences is more likely to recognize the same behavior in a child.

### Associations between the LBC and socio-demographic variables

In the adjusted models, child BMI SDS was predictive of four out of five factors on the LBC problem scale, demonstrating that the questionnaire indeed was able to discriminate obesity-related lifestyle behaviors. Interestingly, the Confidence scale was not associated with any of the studied child and parental characteristics. Likewise, the Dutch validation study [9] was not able to show any associations between child or parent BMI and the Confidence scale. Thus, the results suggest parental confidence is determined by factors other than those examined here, or that confidence is a more stable characteristic, related to personality factors.

No previous study using the LBC has examined in detail the importance of other child and parental socio-demographic variables beyond BMI, such as parental age, gender, education and foreign origin. Our examination showed that while many of these variables had no or small associations with the LBC factors, they were mostly associated with the food related items and with the emotional correlates of being overweight. Child age was only significantly and positively associated with the factor *screen time*, indicating that this new factor may capture behaviors distinct from the physical activity factor.

### Strengths and limitations

This is the largest study on the psychometric properties of the LBC thus far, including a heterogeneous sample of parents of preschool-aged children with normal weight, overweight or obesity, and examining differences between the groups. However, some limitations should be noted. Only half of the parents who received the questionnaire responded; the response rate of 46%, however, is consistent with previous similar studies [13,46]. A further limitation was that weights and heights for both children and parents were self-reported in the school sample; measured values would have increased the validity. Although great efforts were made to include a sample as diverse as possible regarding parental education level, foreign origin and parental and child weight status, the school sample included parents who had higher levels of education and lower levels of overweight/obesity than the general population in Stockholm County. For this reason we included the clinical sample in the analyses, thus making the overall sample more heterogeneous. Moreover, the cross-sectional design of the study does not allow us to draw conclusions about causal effects of child behavior and parenting practices and self-efficacy. Now that the LBC has repeatedly been shown to be a valid instrument, prospective longitudinal interventions can examine whether problematic behaviors in children diminish following intervention, and whether parental self-efficacy can be enhanced. We also encourage researchers from other countries to validate the LBC for greater knowledge about cross-cultural interventions.

## Conclusions

This study has proven the validity of the LBC, a short and user-friendly instrument measuring children’s obesity related problematic behaviors and their parents’ self-efficacy in handling these behaviors, in a large diverse sample of Swedish parents of preschoolers. User-friendly instruments such as the LBC will enable us to learn more about the challenges parents face in preventing and managing childhood obesity, and thus help us tailor effective family based programs.

## Abbreviations

BMI: Body mass index
BMI SDS: Body mass index standard deviation score
CFA: Confirmatory factor analysis
CFI: Comparative fit index
CFQ: Child feeding questionnaire
CN: Concern about child weight (factor of the CFQ)
CONF: Confidence scale (scale of LBC)
EFA: Exploratory factor analysis
EMO: Emotional correlates of being overweight (factor of LBC)
MB: Misbehavior in relation to food (factor of LBC)
MLR: Maximum likelihood with robust standard errors
MN: Monitoring (factor of the CFQ)
LBC: Lifestyle behavior checklist
OE: Overeating (factor of LBC)
PA: Physical activity (factor of LBC)
PR: Parental responsibility (factor of the CFQ)
PCW: Perceived child weight (factor of the CFQ)
PE: Pressure to eat (factor of the CFQ)
PPW: Perceived parent weight (factor of the CFQ)
RMSEA: Root mean square error of approximation
RST: Restriction (factor of the CFQ)
SEM: Structural equation modelling
SRMR: Standardized root mean square residual
ST: Screen time (factor of LBC)
TLI: Tucker-lewis index
